# Metabolomics and Natural-Products Strategies to Study Chemical Ecology in Nematodes

**DOI:** 10.1093/icb/icv077

**Published:** 2015-07-02

**Authors:** Arthur S. Edison, Chaevien S. Clendinen, Ramadan Ajredini, Chris Beecher, Francesca V. Ponce, Gregory S. Stupp

**Affiliations:** *Department of Biochemistry and Molecular Biology and Southeast Center for Integrated Metabolomics, University of Florida, Gainesville, FL 32610-0245, USA;; ^†^IROA Technologies, Ann Arbor, MI, USA;; ^‡^The Scripps Research Institute, Department of Molecular and Experimental Medicine, 10550 North Torrey Pines Road, La Jolla, CA 92037, USA

## Abstract

This review provides an overview of two complementary approaches to identify biologically active compounds for studies in chemical ecology. The first is activity-guided fractionation and the second is metabolomics, particularly focusing on a new liquid chromatography–mass spectrometry-based method called isotopic ratio outlier analysis. To illustrate examples using these approaches, we review recent experiments using *Caenorhabditis elegans* and related free-living nematodes.

## Introduction

This review focuses on a variety of analytical techniques and approaches that can be used to solve biological problems, especially those in chemical ecology and in behavior. As such, we will summarize some of the different technologies that are now available to characterize metabolites and low molecular weight signaling molecules. Each method has its own particular strengths and weaknesses, which we will emphasize by giving specific examples: research that we, and others, have done over the past decade on the model organism *Caenorhabditis elegans* and related nematodes. Even though it is best known as a model organism for genetics (Brenner 1974) and development (Sulston and Horvitz 1977), *C. elegans* is also an ideal animal for chemical studies (Schroeder 2006).

**Fig. 1:**
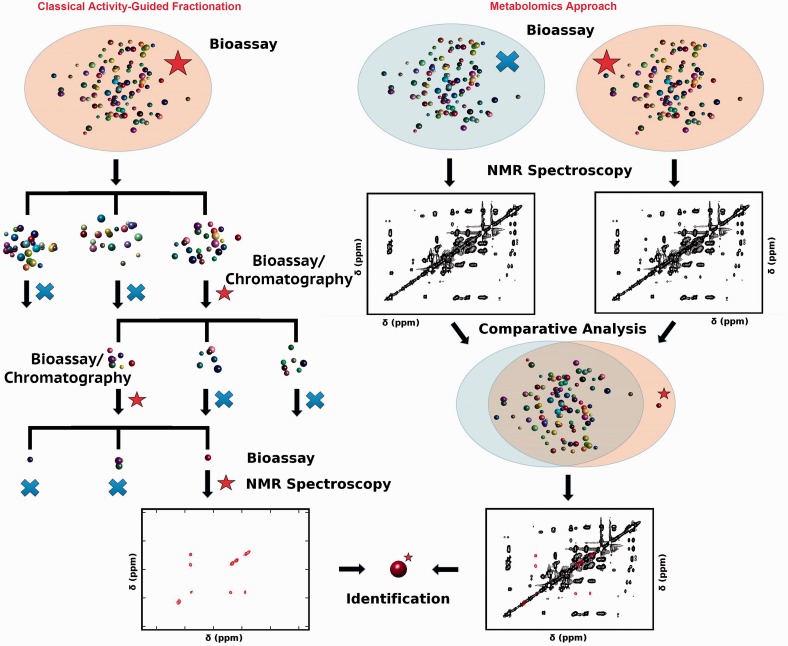
Schematic comparison of two similar strategies to identify active compounds or biomarkers: natural products using activity-guided fraction on the left and metabolomics on the right. Both approaches use similar technology but different steps to get to the same endpoint. Steve Robinette made this figure, which was adapted with permission from Robinette et al. (2012).

It is worth beginning with a brief discussion of two areas of chemical science that are particularly relevant to studies in ecology and behavior. The first is natural products chemistry, which typically starts with a complex mixture from an extract of an organism of interest, partially or fully purifies an active compound(s) using activity-guided fractionation (AGF), and identifies the active compound(s) using nuclear magnetic resonance (NMR) and/or mass spectrometry (MS), which often is coupled to liquid or gas chromatography (LC or GC). The second is metabolomics, which typically starts with complex mixtures from two or more groups that differ by a specific phenotype of interest, collecting NMR or LC–MS/GC–MS data, and comparing the datasets statistically to identify biomarkers that show differences among the groups. The differences and similarities of natural products chemistry and metabolomics are summarized in Fig. 1, which was adapted from a recent review article comparing NMR-based natural products and metabolomics. The conclusion of that article is that the two fields are very closely related and, in fact, are merging in significant ways. Both start with complex mixtures of small-molecule metabolites, use the same analytical technologies, and have as a primary goal the identification of biologically active molecules or biomarkers (Robinette et al. 2012). Similar conclusions have been drawn using LC–MS (Constant and Beecher 1995; Wolfender et al. 2015).

The plan of this review is as follows. The first section focuses on the use of AGF to target specific compounds of interest. This is a time-honored approach that is capable of leading to active compounds, even in low quantities. The drawback is that AGF is also time-consuming and only focuses on relatively few compounds. We then show how metabolomics can be used to obtain chemical information on a more global level, particularly focusing on a new LC–MS technique called isotopic ratio outlier analysis (IROA). We summarize several different approaches that are relevant to chemical ecology studies. Finally, we close with some ideas regarding strategies for integrating phenotypic data with natural products and metabolomics to gain new biological insight on a systems level.

## Activity-guided fractionation

AGF is conceptually simple and extremely useful as a method for discovering molecules with specific functions, but the necessary chromatography can be quite complex. The most important factor in AGF is the assay to measure the activity, which should be sensitive, easy, fast, and reproducible. These can be based on chemistry or biochemistry, such as enzymatic activity, or involve cells or organisms as detectors. A notable assay used male moths in cages as detectors in the purification of the active component of the mating pheromone, as reviewed by Hummel (1984). A more recent and high-tech assay used neuronal recordings of a moth’s antennal lobe combined with GC to identify complex odors associated with plant–pollinator relationships (Riffell et al. 2009). Interestingly, male elephants in a zoo were used to monitor the purification of males’ mating pheromone. We leave it to the imagination of the readers or to the original reference for a description of this purification (Rasmussen et al. 1997). As an evolutionary side-note, moths use the same mating pheromone as a component of their signaling system (Rasmussen et al. 1997).

**Fig. 2:**
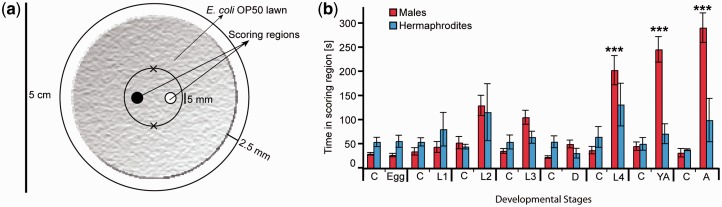
AGF results for the purification of the male-specific mating pheromone from cultures of hermaphroditic *Caenorhabditis elegans*. The assay is shown in (**a**), where about five worms were placed on each of the spots marked with “x” and either buffer control or hermaphrodite-conditioned media were added to the spots in the center. The worms were videotaped and scored by how long they stayed in each spot, which is plotted in (**b**). The data in (b) correspond to material collected from controls (C) and hermaphrodites at different developmental stages (Egg, L1, L2, L3, D = dauer, L4, YA = young adult, and A = adult). Jagan Srinivasan conducted the assays and made the figure, which is reproduced with permission from Srinivasan et al. (2008).

We have used male *C. elegans* as detectors to purify the first mating pheromone in nematodes (Srinivasan et al. 2008). Details are provided in the primary reference, but the general procedure was to isolate large quantities of exudates from liquid cultures of *C. elegans* hermaphrodites and test these for male-specific responses using simple two-spot assays on agar plates, in which one spot has a buffer control and the other spot has a test fraction. We then monitor the time spent by males in each spot (Fig. 2). There are several important considerations when doing one of these assays. First, male and hermaphroditic worms respond to many things, including bacterial food. Therefore, bacteria must be separated from the worms to get a specific and meaningful response. We developed a “worm water” preparation, in which a synchronized population of hermaphrodites at a specific developmental stage was separated from bacteria using a sucrose gradient (Srinivasan et al. 2008; Kaplan et al. 2011; Choe et al. 2012). Then, the worms were allowed to sit in water or a defined buffer, and the supernatant was collected for bioassays.

When purifying an unknown compound, it is important to develop a simple but accurate “accounting” system. Since by definition, we do not know what we are looking for before we find it, we can’t use molarity. When working with worms, we use our own units called “worm equivalents”, for which 1 worm equivalent is defined as the amount of material released by one worm in 1 h. Any convenient and consistent definition will suffice. Using this simple metric, it is easy to identify synergy or loss of activity or to detect other confounding factors.

**Fig. 3:**
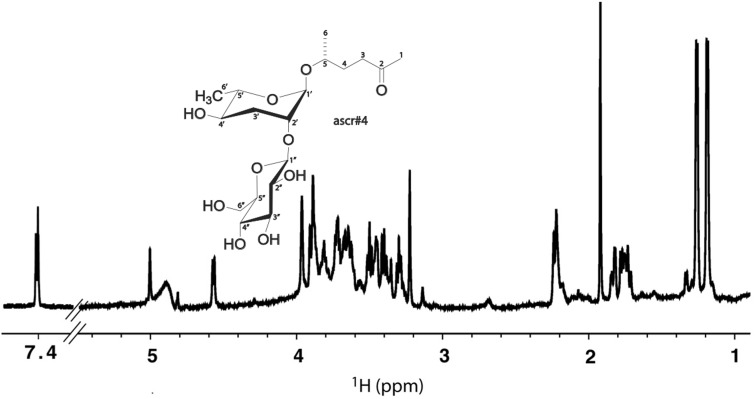
1D ^1^H NMR spectrum from our study to isolate and identify a component of the mating pheromone of *Caenorhabditis elegans* (Srinivasan et al. 2008). This spectrum was collected using one of the most sensitive NMR probes available (Brey et al. 2006). Because the pheromone is present in such low concentrations, this spectrum required about 4 million worm equivalents of material. A male *C. elegans* can detect as little as 2.5 worm equivalents (Kaplan et al. 2011).

We discovered after our initial study that male worms were able to respond to as little as 2.5 worm equivalents, which makes biological sense (Kaplan et al. 2011). It was also humbling to discover that our NMR spectrometer required about 4 million worm equivalents to detect the same pheromone (Fig. 3). This illustrates possibly the most important advantage of AGF over the metabolomics experiments described below: with a very sensitive bioassay like a male worm or moth, it is possible to detect the important components at concentrations that would be entirely missed with analytical instruments.

**Fig. 4:**
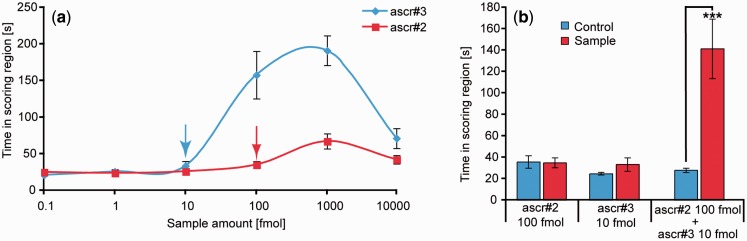
Dose response curves (**a**) and synergy (**b**) of the attraction of male *Caenorhabditis elegans* to two different mating pheromones. The assays were as described in Fig. 2. Panel (a) shows that there can be points of maximal attraction and that caution needs to be used to avoid adding too much material in a bioassay. The blue and red arrows in (a) indicate the concentrations tested in (b), which shows the results of adding together two pheromones at concentrations that each alone would produce no activity but that together produce a large response. All of these very interesting biological stories can cause great confusion in an AGF study. Reproduced from Srinivasan et al. (2008).

Male worms may be extremely sensitive, but this can add other potential complications to an AGF. First, many response curves for pheromones are bell-shaped, with maximal responses tuned to appropriate concentrations and falling off at higher or lower concentrations, presumably stopping mate-finding behavior when a mate is located (Fig. 4a). It is important at the start of a new AGF study to carefully examine the crude extract (e.g., “worm water” for many of our studies) for activity at a wide-range of concentrations (e.g., worm equivalents) to avoid putting too much material onto the assay. However, this can also backfire, because if there are different compounds that cause the opposite response or block the primary response, the starting material may not work. A good example of teasing apart opposite responses in behavioral assays was an assay developed by Srinivasan et al. (2012) to characterize simultaneous attractive and repulsive responses to a mixture of two related pheromones in *C. elegans*. Briefly, three concentric scoring regions were defined, and the time spent in the center was compared with the time spent in the outer circles.

One of the major complications of AGF is a synergetic effect of different metabolites responsible for the activity of the crude extract. In our study of the identification of males’ mating pheromone in *C. elegans*, we lost all activity at a key step in the fractionation (Srinivasan et al. 2008). The loss of activity could be caused by several factors, including degradation of the molecule(s), loss of the molecule(s) on the column, or synergy. Combining all the fractions and testing for activity, which will return if two or more compounds are acting synergistically, can rule out degradation or loss. With synergy, AGF gets more complicated. The evidence of synergy in that study is shown in Fig. 4b. Often a good start is to identify a fraction that is necessary but not sufficient for activity. This will yield at least one component, which can then be added to other fractions for additional bioassays.

In summary, AGF is a powerful but potentially very time-consuming way to identify biologically significant molecules. It can often reveal activity at concentrations that would be too low to measure with analytical instrumentation, and since it is always directed to the activity of interest, positive results will be relevant to the study. Synergy, compounds with masking or opposing activities, and bell-shaped responses can all complicate AGF. We have conducted two large AGF identifications of nematodes’ mating pheromones, and they both required several years of work to complete (Srinivasan et al. 2008; Choe et al. 2012); they are definitely not high-throughput!

## Metabolomic strategies

The right side of Fig. 1 summarizes a metabolomics approach to the identification of biomarkers. As illustrated, the end-point of a metabolomics and natural-products (AGF) study can be the same, but the mechanism and steps to get there are quite different. In metabolomics, the laborious step of AGF is eliminated. This can be wonderful news to anyone who has conducted an AGF study. There are some important differences in terms of overall design and outcomes of experiments between AGF and metabolomics.

The first is replicates of samples. Historically, AGF studies have not focused on obtaining large numbers of unique biological replicates, but in most cases many different samples need to be generated over the course of a study for subsequent steps in a purification scheme. In contrast, metabolomics is completely dependent upon statistical analysis. Therefore, a well-controlled design of the study, including appropriate numbers of replicates, is critical. It is always important in a metabolomics study to have discussions with an expert statistician.

Another important difference between AGF and metabolomics is the point at which analytical sensitivity becomes a major factor. Both approaches use the same types of MS and NMR instruments, but the reliance on the instruments is significantly different. In the example described above, male *C. elegans* were able to detect a pheromone produced by about 2.5 worm equivalents (Kaplan et al. 2011), while an outstanding NMR system (Brey et al. 2006) needed about 4 million worm equivalents of the same material. Based on knowledge gained from subsequent studies, we estimate that a sensitive LC–MS instrument would need about 10,000 worm equivalents for reliable detection of the same material (Stupp et al. 2013). The critical point here is that if we were relying entirely on NMR and MS instruments, as in the case in metabolomics, we would not find an active compound that is below our detection limits. However, with AGF, we know something of interest to the male worms is there, even if we need to produce a lot of material and concentrate it for NMR and MS. Thus, with metabolomics it is critical to always remember that the analytical instrumentation will define the number and concentrations of metabolites detected. Important biologically active compounds at concentrations too low to measure will be completely invisible in a metabolomics study.

One important distinction to make for metabolomics is “targeted” versus “untargeted” or “global” metabolomics. A full discussion of these is beyond the scope of this review, but excellent review articles have been published (Fiehn 2002; Dunn et al. 2011). In many ways, AGF could be considered a type of targeted metabolomics, for which the goal is to identify and quantify a specific compound based on activity. Targeted assays generally refer to classes such as “amino acids”, “organic acids”, but by using a biological detector like a male worm, a target class could also be “the group of compounds that causes males to be attracted”. Global metabolomics has several compelling advantages over targeted studies if an unbiased view of a given system (e.g., hypothesis-generation) is required. There are several different types of metabolomics experiments and general approaches to complex mixtures. We have recently reviewed and contrasted some of the methods used in NMR (Robinette et al. 2012). Here, we briefly describe a new metabolomics experiment that we think has some particular advantages for studies in chemical ecology.

As suggested above in the comparison of how many worm equivalents were needed to characterize a mating pheromone of *C. elegans*, NMR is less sensitive than MS. NMR typically requires about 100 nanomoles of material, but significant improvements with specialized technology can lower that to the just a few nanomoles (Dalisay et al. 2009; Molinski 2010; Ramaswamy et al. 2013). The major advantage of NMR is that it provides atom-specific information that is necessary for the full identification of an unknown molecule. NMR is also very reproducible, in part because the sample is in a tube that is not in contact with the instrument. Finally, NMR can be quantitative when the spectroscopist allows for full relaxation between scans. In contrast, MS is extremely sensitive, requiring much less material than NMR. For targeted analyses of known molecules, MS is generally much more efficient than NMR. On the other hand, LC–MS data can have many artifacts, and reproducibility is challenging, in part because the sample is in contact both with the chromatographic column and the detector in the MS instrument. MS data provide the mass/charge of each detected ion, and some instruments are capable tandem MS (MS^n^), which fragments ions that can be analyzed *de novo* or matched to databases for more definitive identification. Several reviews are available that expand on these basic introductory concepts (Katajamaa and Oresic 2007; Moco et al. 2007; Scalbert et al. 2009; Edison and Schroeder 2010; Wolfender et al. 2013).

## Isotopic ratio outlier analysis

**Fig. 5:**
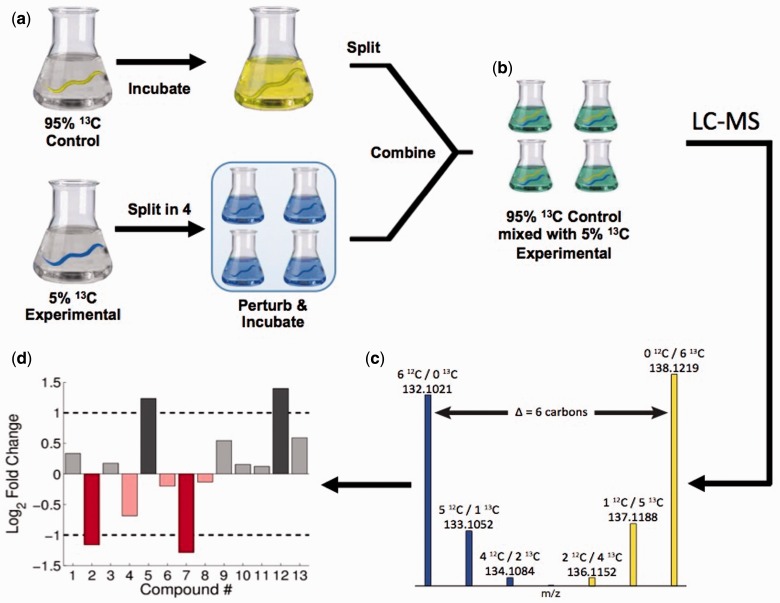
IROA method: (**a**) Experimental and control groups of worms are isotopically labeled at 5% or 95% ^13^C and grown to the stage of young adults. The experimental group is split into four replicates and is perturbed, while the control group is not split. After incubation, the control group is split into four replicates, and each replicate is mixed 1:1 with an experimental replicate (**b**) for uniform preparation of samples and for LC–MS analysis. (**c**) Biological compounds are easily distinguished from artifacts by the recognizable pattern caused by the isotopic enrichment. (**d**) Using automated software, the fold-changes for all detected biological compounds can be determined. Reproduced with permission from Stupp et al. (2013).

IROA is a relatively new LC–MS-based experiment that we have used in experiments on metabolomics in *C. elegans* (Stupp et al. 2013). It has been reviewed (de Jong and Beecher 2012), so here we will focus on its utility for chemical ecology. In the basic IROA experiment, two different populations are compared, one that has been labeled with 5% ^13^C and the other with 95% ^13^C. ^13^C is a stable isotope that not only is useful for MS experiments but also is an excellent NMR nucleus, allowing for some combined analysis. The basic IROA workflow is shown in Fig. 5. There are several advantages of IROA for LC–MS metabolomics. First, the patterns of peaks created by the isotope labeling are easy to detect by a computer, greatly reducing artifacts. Second, the number of carbon atoms is known exactly for each MS peak, allowing for very efficient determination of molecular formulas. Finally, the relative quantities of the 5% and 95% channels are easily quantified. The result is that hundreds to thousands of peaks can be reliably detected and quantified. It is relatively easy to assign peaks to a molecular formula, but it is a greater challenge to figure out the true identity of all the peaks.

There are many experiments that one could do with IROA. We will illustrate these with examples from nematodes, but virtually any system that can be labeled can be studied. For example, wild isolates of *C. elegans,* or other free-living species, could be collected and labeled with 5% ^13^C using labeled bacterial food. These could be compared with a common reference material of the laboratory wild-type (N2) strain that had been labeled with 95% ^13^C. With a very large batch of reference material, many different pairwise experiments can be conducted, and differences between wild isolates and N2 can be determined. These metabolic differences can then be compared with behavioral differences to correlate molecules or pathways with the phenotype. Another example would be to label a given strain with both 5% and 95% ^13^C and then add an unlabeled pathogen or other perturbation to the 5% channel. In this scenario, the global metabolic responses to the perturbation can be measured. Finally, in an experiment called “phenotypic IROA” (de Jong and Beecher 2012), the 5% ^13^C channel can be replaced by natural abundance ^13^C, which simply changes the pattern of isotopic distribution on the ^12^C side (blue peaks in Fig. 5). Some information is lost from the traditional IROA experiment, but with this it is possible to compare different species even if some cannot be cultured. For example, parasitic nematodes are notoriously difficult to culture, but one could compare these to a reference 95% ^13^C labeled *C. elegans*. It is important to note that in this situation, the experiment is more similar to a targeted study in which the targeted compounds are all labeled compounds from the reference strain, because they are the only ones that can be detected.

## Conclusion

We have attempted to illustrate different approaches that we have used to study chemical ecology in complex systems. The examples of nematodes follow from our own work and interests, but many different types of systems can be studied using similar experiments. There is no “perfect” approach, and in practice a combination of a natural products-based AGF and metabolomics is perhaps ideal. To find out the molecular basis of a specific behavior, a good starting point would be a simple and reliable bioassay for that behavior, followed by an AGF of the active compound(s). In parallel or sequentially, a metabolomics experiment could be developed to look more globally at the problem, either to avoid bias or to capture other compounds that may be relevant. A nice example of a combined approach is illustrated by the identification of both a male-specific and a female-specific mating pheromone in *Panagrellus redivivus* (Choe et al. 2012). In that study, the Edison and Sternberg laboratories conducted AGF on a mixed culture of worms and continued until gender-specific components were separated chromatographically. Two different ascarosides were discovered, ascr#1 that attracts males and dhas#18 that attracts females. Simultaneously, the Schroeder laboratory conducted a metabolomics-type experiment that used LC–MS to detect all ascarosides made by the species. They discovered ascr#1 and dhas#18, along with several other ascarosides not found in the AGF study. However, upon testing of the other ascarosides, none of them had significant activity in gender-specific attraction, suggesting other unknown functions (Choe et al. 2012). This study nicely illustrates the power both of natural products AGF and of metabolomics, which often can be used together to approach a question from two sides of the same coin (Robinette et al. 2012).

## Funding

This work was supported by the Southeast Center for Integrated Metabolomics (NIH 1U24DK097209).
